# An integrative multi-omics analysis to identify candidate DNA methylation biomarkers related to prostate cancer risk

**DOI:** 10.1038/s41467-020-17673-9

**Published:** 2020-08-06

**Authors:** Lang Wu, Yaohua Yang, Xingyi Guo, Xiao-Ou Shu, Qiuyin Cai, Xiang Shu, Bingshan Li, Ran Tao, Chong Wu, Jason B. Nikas, Yanfa Sun, Jingjing Zhu, Monique J. Roobol, Graham G. Giles, Hermann Brenner, Esther M. John, Judith Clements, Eli Marie Grindedal, Jong Y. Park, Janet L. Stanford, Zsofia Kote-Jarai, Christopher A. Haiman, Rosalind A. Eeles, Wei Zheng, Jirong Long, The PRACTICAL consortium, Rosalind A. Eeles, Brian E. Henderson, Christopher A. Haiman, Zsofia Kote-Jarai, Fredrick R. Schumacher, Douglas Easton, Sara Benlloch, Ali Amin Al Olama, Kenneth Muir, Sonja I. Berndt, David V. Conti, Fredrik Wiklund, Stephen Chanock, Susan M. Gapstur, Victoria L. Stevens, Catherine M. Tangen, Jyotsna Batra, Judith Clements, Henrik Gronberg, Nora Pashayan, Johanna Schleutker, Demetrius Albanes, Stephanie Weinstein, Alicja Wolk, Catharine West, Lorelei Mucci, Géraldine Cancel-Tassin, Stella Koutros, Karina Dalsgaard Sorensen, Eli Marie Grindedal, David E. Neal, Freddie C. Hamdy, Jenny L. Donovan, Ruth C. Travis, Robert J. Hamilton, Sue Ann Ingles, Barry S. Rosenstein, Yong-Jie Lu, Graham G. Giles, Adam S. Kibel, Ana Vega, Manolis Kogevinas, Kathryn L. Penney, Jong Y. Park, Janet L. Stanford, Cezary Cybulski, Børge G. Nordestgaard, Hermann Brenner, Christiane Maier, Jeri Kim, Esther M. John, Manuel R. Teixeira, Susan L. Neuhausen, Kim De Ruyck, Azad Razack, Lisa F. Newcomb, Marija Gamulin, Radka Kaneva, Nawaid Usmani, Frank Claessens, Paul A. Townsend, Manuela Gago Dominguez, Monique J. Roobol, Florence Menegaux, Kay-Tee Khaw, Lisa Cannon-Albright, Hardev Pandha, Stephen N. Thibodeau, David J. Hunter, William J. Blot, Elio Riboli, CRUK Consortium, Rosalind A. Eeles, Zsofia Kote-Jarai, Catharine West, David E. Neal, Freddie C. Hamdy, Jenny L. Donovan, Ruth C. Travis, Elio Riboli, BPC3 Consortium, Brian E. Henderson, Christopher A. Haiman, Fredrick R. Schumacher, Sonja I. Berndt, Stephen Chanock, Susan M. Gapstur, Victoria L. Stevens, Demetrius Albanes, Stephanie Weinstein, Lorelei Mucci, Stella Koutros, Ruth C. Travis, Kathryn L. Penney, David J. Hunter, Elio Riboli, CAPS Consortium, Fredrik Wiklund, Henrik Gronberg, PEGASUS Consortium, Sonja I. Berndt, Stephen Chanock, Demetrius Albanes, Stephanie Weinstein, Stella Koutros

**Affiliations:** 1 Cancer Epidemiology Division, Population Sciences in the Pacific Program, University of Hawaii Cancer Center, University of Hawaii at Manoa, Honolulu, HI USA; 2 Division of Epidemiology, Department of Medicine, Vanderbilt Epidemiology Center, Vanderbilt-Ingram Cancer Center, Vanderbilt University Medical Center, Nashville, TN USA; 3 Department of Molecular Physiology & Biophysics, Vanderbilt University, Nashville, TN USA; 4 Vanderbilt Genetics Institute, Vanderbilt University Medical Center, Nashville, TN USA; 5 Department of Biostatistics, Vanderbilt University Medical Center, Nashville, TN USA; 6 Department of Statistics, Florida State University, Tallahassee, FL USA; 7 Research & Development, Genomix Inc, Minneapolis, MN USA; 8 College of Life Science, Longyan University, Longyan, Fujian P. R. China; 9 Department of Urology, Erasmus University Medical Center, Rotterdam, The Netherlands; 10 Centre for Epidemiology and Biostatistics, Melbourne School of Population and Global Health, University of Melbourne, 207 Bouverie St, Melbourne, VIC 3010 Australia; 11 Cancer Epidemiology & Intelligence Division, Cancer Council Victoria, 615 St Kilda Rd, Melbourne, VIC 3004 Australia; 12 Division of Clinical Epidemiology and Aging Research, German Cancer Research Center (DKFZ), Heidelberg, Germany; 13 German Cancer Consortium (DKTK), German Cancer Research Center (DKFZ), Heidelberg, Germany; 14 Division of Preventive Oncology, German Cancer Research Center (DKFZ) and National Center for Tumor Diseases (NCT), Heidelberg, Germany; 15 Department of Medicine (Oncology) and Stanford Cancer Institute, Stanford University School of Medicine, Stanford, CA USA; 16 Australian Prostate Cancer Research Centre-QLD, Institute of Health and Biomedical Innovation and School of Biomedical Science, Queensland University of Technology, Brisbane, QLD Australia; 17 Translational Research Institute, Brisbane, QLD Australia; 18 Department of Medical Genetics, Oslo University Hospital, Oslo, Norway; 19 Department of Cancer Epidemiology, Moffitt Cancer Center, Tampa, FL USA; 20 Division of Public Health Sciences, Fred Hutchinson Cancer Research Center, Seattle, WA USA; 21 Department of Epidemiology, School of Public Health, University of Washington, Seattle, WA USA; 22 Division of Genetics and Epidemiology, The Institute of Cancer Research, and The Royal Marsden NHS Foundation Trust, London, UK; 23 Department of Preventive Medicine, University of Southern California, Los Angeles, CA USA; 24 Department of Population and Quantitative Health Sciences, Case Western Reserve University, Cleveland, OH USA; 25 Seidman Cancer Center, University Hospitals, Cleveland, OH USA; 26 Centre for Cancer Genetic Epidemiology, Department of Public Health and Primary Care, University of Cambridge, Strangeways Research Laboratory, Cambridge, UK; 27 University of Cambridge, Department of Clinical Neurosciences, Cambridge, UK; 28 Division of Population Health, Health Services Research and Primary Care, University of Manchester, Oxford Road, Manchester, UK; 29 Warwick Medical School, University of Warwick, Coventry, UK; 30 Division of Cancer Epidemiology and Genetics, National Cancer Institute, NIH, Bethesda, MD USA; 31 Department of Medical Epidemiology and Biostatistics, Karolinska Institute, Stockholm, Sweden; 32 Epidemiology Research Program, American Cancer Society, 250 Williams Street, Atlanta, GA USA; 33 SWOG Statistical Center, Fred Hutchinson Cancer Research Center, Seattle, WA USA; 34 Institute of Health and Biomedical Innovation and School of Biomedical Sciences, Queensland University of Technology, Brisbane, QLD 4059 Australia; 35 University College London, Department of Applied Health Research, London, UK; 36 Centre for Cancer Genetic Epidemiology, Department of Oncology, University of Cambridge, Strangeways Laboratory, Cambridge, UK; 37 Institute of Biomedicine, Kiinamyllynkatu 10, FI-20014 University of Turku, Turku, Finland; 38 Tyks Microbiology and Genetics, Department of Medical Genetics, Turku University Hospital, PO Box 52, 20521 Turku, Finland; 39 Division of Nutritional Epidemiology, Institute of Environmental Medicine, Karolinska Institutet, Solna, Sweden; 40 Department of Surgical Sciences, Uppsala University, Uppsala, Sweden; 41 Division of Cancer Sciences, University of Manchester, Manchester Academic Health Science Centre, Radiotherapy Related Research, Manchester NIHR Biomedical Research Centre, The Christie Hospital NHS Foundation Trust, Manchester, UK; 42 Department of Epidemiology, Harvard School of Pubic Health, Boston, MA USA; 43 CeRePP, Tenon Hospital, Paris, France; 44 UPMC Sorbonne Universites, GRC N°5 ONCOTYPE-URO, Tenon Hospital, 4 rue de la Chine, Paris, France; 45 Department of Molecular Medicine, Aarhus University Hospital, Aarhus, Denmark; 46 Department of Clinical Medicine, Aarhus University, Aarhus, Denmark; 47 University of Cambridge, Department of Oncology, Addenbrooke’s Hospital, Cambridge, UK; 48 Cancer Research UK Cambridge Research Institute, Li Ka Shing Centre, Cambridge, UK; 49 Nuffield Department of Surgical Sciences, Faculty of Medical Science, University of Oxfordm, John Radcliffe Hospital, Oxford, UK; 50 School of Social and Community Medicine, University of Bristol, Bristol, UK; 51 Cancer Epidemiology Unit, Nuffield Department of Population Health University of Oxford, Oxford, UK; 52 Department of Surgical Oncology, Princess Margaret Cancer Centre, Toronto, Canada; 53 Department of Radiation Oncology, Icahn School of Medicine at Mount Sinai, New York, NY USA; 54 Department of Genetics and Genomic Sciences, Icahn School of Medicine at Mount Sinai, New York, NY USA; 55 Centre for Molecular Oncology, Barts Cancer Institute, Queen Mary University of London, John Vane Science Centre, London, UK; 56 Division of Urologic Surgery, Brigham and Womens Hospital, Boston, MA USA; 57 Fundación Pública Galega de Medicina Xenómica-SERGAS, Grupo de Medicina Xenómica, CIBERER, IDIS, Santiago de Compostela, Spain; 58 Centre for Research in Environmental Epidemiology (CREAL), Barcelona Institute for Global Health (ISGlobal), Barcelona, Spain; 59 CIBER Epidemiología y Salud Pública (CIBERESP), Madrid, Spain; 60 IMIM (Hospital del Mar Research Institute), Barcelona, Spain; 61 Universitat Pompeu Fabra (UPF), Barcelona, Spain; 62 Channing Division of Network Medicine, Department of Medicine, Brigham and Women’s Hospital/Harvard Medical School, Boston, MA USA; 63 International Hereditary Cancer Center, Department of Genetics and Pathology, Pomeranian Medical University, Szczecin, Poland; 64 Faculty of Health and Medical Sciences, University of Copenhagen, Copenhagen, Denmark; 65 Department of Clinical Biochemistry, Herlev and Gentofte Hospital, Copenhagen University Hospital, Herlev, Denmark; 66 Institute for Human Genetics, University Hospital Ulm, Ulm, Germany; 67 The University of Texas M. D. Anderson Cancer Center, Department of Genitourinary Medical Oncology, Houston, TX USA; 68 Department of Genetics, Portuguese Oncology Institute of Porto, Porto, Portugal; 69 Biomedical Sciences Institute (ICBAS), University of Porto, Porto, Portugal; 70 Department of Population Sciences, Beckman Research Institute of the City of Hope, Duarte, CA USA; 71 Ghent University, Faculty of Medicine and Health Sciences, Basic Medical Sciences, Gent, Belgium; 72 Department of Surgery, Faculty of Medicine, University of Malaya, Kuala Lumpur, Malaysia; 73 Department of Urology, University of Washington, Seattle, WA USA; 74 Institute of Human Genetics, University Medical Center Hamburg-Eppendorf, Hamburg, Germany; 75 Molecular Medicine Center, Department of Medical Chemistry and Biochemistry, Medical University, Sofia, Bulgaria; 76 Department of Oncology, Cross Cancer Institute, University of Alberta, Edmonton, Alberta Canada; 77 Division of Radiation Oncology, Cross Cancer Institute, Edmonton, Alberta Canada; 78 Molecular Endocrinology Laboratory, Department of Cellular and Molecular Medicine, KU Leuven, Leuven, Belgium; 79 Division of Cancer Sciences, Manchester Cancer Research Centre, Faculty of Biology, Medicine and Health, Manchester Academic Health Science Centre, NIHR Manchester Biomedical Research Centre, Health Innovation Manchester, Univeristy of Manchester, Manchester, UK; 80 Genomic Medicine Group, Galician Foundation of Genomic Medicine, Instituto de Investigacion Sanitaria de Santiago de Compostela (IDIS), Complejo Hospitalario Universitario de Santiago, Servicio Galego de Saúde, SERGAS, Santiago De Compostela, Spain; 81 University of California San Diego, Moores Cancer Center, La Jolla, CA USA; 82 Cancer & Environment Group, Center for Research in Epidemiology and Population Health (CESP), INSERM, University Paris-Sud, University Paris-Saclay, Villejuif, France; 83 Clinical Gerontology Unit, University of Cambridge, Cambridge, UK; 84 Division of Genetic Epidemiology, Department of Medicine, University of Utah School of Medicine, Salt Lake City, UT USA; 85 George E. Wahlen Department of Veterans Affairs Medical Center, Salt Lake City, UT USA; 86 The University of Surrey, Guildford, Surrey, UK; 87 Department of Laboratory Medicine and Pathology, Mayo Clinic, Rochester, MN USA; 88 Program in Genetic Epidemiology and Statistical Genetics, Department of Epidemiology, Harvard T.H. Chan School of Public Health, Boston, MA USA; 89 International Epidemiology Institute, Rockville, MD USA; 90 Department of Epidemiology and Biostatistics, School of Public Health, Imperial College London, London, SW7 2AZ UK

**Keywords:** Cancer epidemiology, Cancer genetics, Genetics

## Abstract

It remains elusive whether some of the associations identified in genome-wide association studies of prostate cancer (PrCa) may be due to regulatory effects of genetic variants on CpG sites, which may further influence expression of PrCa target genes. To search for CpG sites associated with PrCa risk, here we establish genetic models to predict methylation (N = 1,595) and conduct association analyses with PrCa risk (79,194 cases and 61,112 controls). We identify 759 CpG sites showing an association, including 15 located at novel loci. Among those 759 CpG sites, methylation of 42 is associated with expression of 28 adjacent genes. Among 22 genes, 18 show an association with PrCa risk. Overall, 25 CpG sites show consistent association directions for the methylation-gene expression-PrCa pathway. We identify DNA methylation biomarkers associated with PrCa, and our findings suggest that specific CpG sites may influence PrCa via regulating expression of candidate PrCa target genes.

## Abstract (web summary)

Genome wide association studies have identified multiple loci associated with risk of developing prostate cancer but the functional significance of many of these are unknown. Here, after generating models to predict methylation, the authors identify CpG methylation sites associated with prostate cancer.

## Introduction

Prostate cancer (PrCa) is the second most frequently diagnosed malignancy among men and the fifth leading cause of cancer death worldwide^1^. Its survival rate is relatively high for localized stage disease, but decreases substantially for metastatic disease^2^. Effective strategies are critical for risk assessment, screening, and early detection of PrCa, aimed at decreasing its public health burden. Although prostate-specific antigen (PSA) has demonstrated efficacy for detecting PrCa early^3,4^, there lacks a clear cutoff point for PSA with high sensitivity and specificity^5–7^. The benefits of PSA screening for reducing PrCa mortality remains controversial^8–10^. Furthermore, there are adverse effects, such as overdiagnosis^11^. Therefore, additional effective biomarkers are needed for risk assessment and early detection of PrCa.

Aligned with findings of a crucial role for DNA methylation in PrCa development^12^, research has identified several methylation markers to be potentially associated with PrCa risk, such as methylation at *GSTP1*, *CDKN2A*, *DNMT3B*, *SCGB3A1*, and *HIF3A*^12–16^. However, most prior studies have assessed only a couple of candidates. Recent emerging studies profiling genome-wide methylation usually included a relatively small number of subjects^17^, resulting in inadequate power for the identification of associated methylation biomarkers. Besides these limitations, there are a number of biases commonly encountered in conventional epidemiologic studies, including selection bias, uncontrolled confounding, and reverse causation, that make it difficult to determine whether the identified associated markers are causally associated with PrCa.

One strategy to reduce some of these biases is to use genetic variants to develop an instrument to assess the association between DNA methylation and PrCa. Such an approach is based on the principle of the random assortment of alleles from parents to offspring during gamete formation, and thus a genetically determined proportion of DNA methylation levels should be less susceptible to selection bias and reverse causation in principal. Research has shown that a large portion of CpG sites have high heritability^18,19^. Genome-wide association studies (GWAS) have also identified a large number of genetic loci associated with DNA methylation levels^20,21^. Many of these genetic variants could potentially serve as strong instrumental variables for evaluating associations between DNA methylation and PrCa risk in an adequately powered study.

Besides a potential utility in improving PrCa risk assessment, the identification of promising DNA methylation markers using a design of genetic instruments may also contribute to understanding of the genetics and etiology of PrCa. Epidemiological research provides strong support for a genetic predisposition to PrCa^22,23^. To date, GWAS have identified ~150 genetic loci for PrCa^24–26^. However, together these variants explain <30% of the familial relative risk, and the underlying biological mechanisms for a majority of the identified loci remain unclear^24^. Recently, we performed a large transcriptome-wide association study (TWAS) of PrCa, in which we identified multiple associations between genetically predicted gene expression and PrCa risk^27^. Interestingly, many of the associated genes were identified to be candidate target genes of GWAS-identified risk SNPs^27^. Aligned with the recognized role of DNA methylation in regulating gene expression, we hypothesize that some GWAS-identified risk SNPs may regulate expression of their target genes through influencing DNA methylation levels. In this study, we perform a large integrative multi-omics analysis involving data of genomics, methylomics, and transcriptomics aiming to uncover novel CpG sites and genes that may contribute to PrCa development.

## Results

### DNA methylation prediction models

Using FHS data, we were able to build DNA methylation prediction models for 223,959 CpG sites, of which 81,432 showed a prediction performance (*R*^2^) of at least 0.01 (≥10% correlation between predicted and measured DNA methylation levels). For 77,243 of those CpG sites, there were no SNPs within the binding site. Interestingly, there tended to be positive weak correlations between methylation prediction model performance and number of input variants within the 2-MB window of each CpG site (Pearson correlation coefficient 0.03, *P* = 1.60 × 10^−13^; Spearman correlation coefficient 0.02, *P* = 1.43 × 10^−6^). We further applied these 77,243 models to the genetic data in WHI and evaluated their performance by comparing predicted methylation levels with measured levels. Overall, DNA methylation that could be predicted well in FHS also tended to be predicted well in WHI (a correlation coefficient of 0.96 for *R*^2^ in two datasets; Supplementary Fig. 1). These 77,243 CpG sites were selected for analyses for their associations between predicted DNA methylation and PrCa risk.

### Associations of genetically predicted methylation with PrCa

**Fig. 1:**
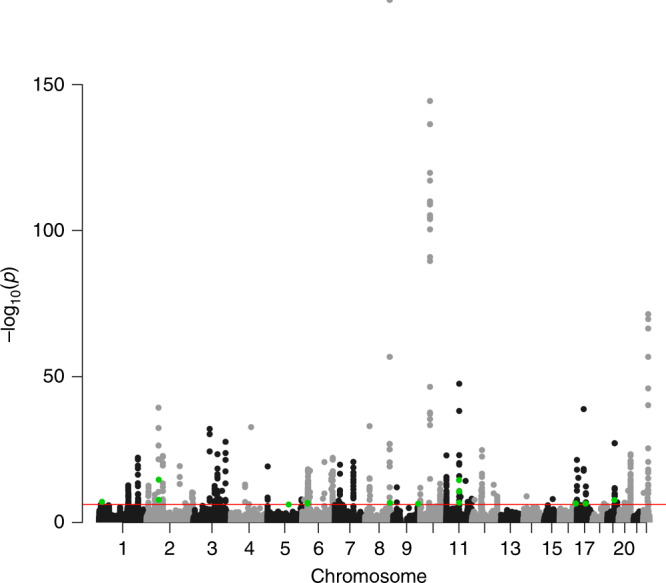
A Manhattan plot of the association results from the prostate cancer methylome-wide association study using S-PrediXcan. The red line represents *P* = 6.47 × 10^−7^ (Bonferroni correction of 77,243 tests (0.05/77,243)). Each dot represents the genetically predicted DNA methylation of one specific CpG site. The *x* axis represents the genomic position of the corresponding CpG site, and the *y* axis represents the negative logarithm of the association *P* value. CpG sites at novel loci were highlighted with green color. Two-sided test was conducted.

Of the 77,243 CpG sites tested, genetically predicted DNA methylation of 759 located at 82 genomic loci were associated with PrCa risk after Bonferroni correction (*P* ≤ 6.47 × 10^−7^) (Table 1; Supplementary Table 1 and Supplementary Data 1; Manhattan plot in Fig. 1). This included 15 located at 10 genomic loci that were more than 500 kb away from any PrCa risk variant identified in GWAS or fine-mapping studies (Table 1). An association between a higher DNA methylation level and increased PrCa risk was detected for cg18800143, cg07645299, cg12627844, cg16397176, cg11562153, cg13866093, cg00444740, cg20100049, cg22370235, cg04739953, cg01715842, and cg23397578. Conversely, an inverse association between methylation level and PrCa risk was identified for cg24388424, cg06836406, and cg13230424. Of these 15 CpG sites at novel loci, after conditioning on the near PrCa risk variant, the associations of genetically predicted DNA methylation levels for four CpG sites (cg18800143, cg16397176, cg06836406, and cg13230424) remained at *P* ≤ 6.47 × 10^−7^ (Table 1).

**Table 1:** Fifteen novel methylation-prostate cancer associations for CpG sites located at genomic loci at least 500 kb away from any known prostate cancer risk variant^a^.

CpG site	Chr	Position (build37)	Classification	*R*^2b^	OR (95% CI)^c^	*P* value^d^	risk SNP	Distance to the risk SNP (kb)	*P* value after adjusting for risk SNP^e^
cg18800143	1	16393791	Intronic	0.10	1.12 (1.07–1.17)	7.56 × 10^−8^	rs636291	5837.7	**7.07** × **10**^**−9**^
cg07645299	2	63991864	Intergenic	0.01	1.49 (1.30–1.71)	1.58 × 10^−8^	rs58235267	714.0	0.80
cg12627844	2	64245000	Intronic	0.03	1.38 (1.28–1.50)	1.98 × 10^−15^	rs58235267	967.2	0.61
cg16397176	5	110899314	ncRNA_intronic	0.05	1.15 (1.09–1.22)	6.42 × 10^−7^	rs10793821	22936.9	**6.25** × **10**^**−7**^
cg11562153	6	28493500	Upstream	0.04	1.22 (1.13–1.31)	1.57 × 10^−7^	rs7767188	1580.3	1.56 × 10^−4^
cg13866093	6	28502727	UTR3	0.05	1.14 (1.09–1.20)	2.09 × 10^−7^	rs7767188	1571.0	3.26 × 10^−5^
cg24388424	6	28565403	Intronic	0.01	0.78 (0.71–0.86)	3.31 × 10^−7^	rs7767188	1508.4	1.08 × 10^−5^
cg00444740	8	129162178	Upstream	0.02	1.21 (1.13–1.30)	1.55 × 10^−7^	rs7837688	622.8	1.01 × 10^−3^
cg06836406	9	130461544	Intergenic	0.02	0.79 (0.72−0.86)	3.55 × 10^−7^	rs1182	2114.5	**1.74** × **10**^**−7**^
cg20100049	11	67979188	Intronic	0.02	1.30 (1.22–1.39)	2.79 × 10^−15^	rs11228565	999.4	2.44 × 10^−4^
cg22370235	11	68451852	Upstream	0.02	1.29 (1.17–1.41)	1.50 × 10^−7^	rs11228565	526.7	0.37
cg04739953	11	68451858	Upstream	0.01	1.62 (1.41–1.87)	2.06 × 10^−11^	rs11228565	526.7	0.15
cg01715842	16	85045600	Upstream	0.47	1.05 (1.03–1.07)	2.95 × 10^−7^	rs199737822	2866.7	NA
cg13230424	17	45930033	Intronic	0.05	0.87 (0.82–0.91)	3.16 × 10^−7^	rs138213197	875.7	**5.74** × **10**^**−8**^
cg23397578	19	37742925	ncRNA_exonic	0.01	1.40 (1.24–1.57)	1.81 × 10^−8^	rs8102476	992.7	1.57 × 10^−3^

*NA* not available. Bold values represent that these association *p* values remain largely unchanged after adjusting for risk SNP.

^a^Risk SNPs identified in previous GWAS or fine-mapping studies.

^b^*R*^2^: model prediction performance (*R*^2^) derived using FHS data.

^c^OR (odds ratio) and CI (confidence interval) per one standard deviation increase in genetically predicted DNA methylation.

^d^*P* value: derived from association analyses of 79,194 cases and 61,112 controls (two-sided); associations with *P* ≤ 6.47 × 10^−7^ based on Bonferroni correction of 77,243 tests (0.05/77,243) are shown.

^e^Using COJO method.

For the remaining 744 CpG sites located at known PrCa risk loci (Supplementary Table 1 and Supplementary Data 1), after conditioning on the adjacent PrCa risk SNP, an association at *P* ≤ 6.47 × 10^−7^ persisted for 63 CpG sites (Supplementary Table 1). This suggests that the associations of these 63 CpG sites with PrCa risk are potentially independent of the PrCa risk SNPs identified in GWAS or fine-mapping studies (Supplementary Table 1). For the other 681 CpG sites, their associations with PrCa risk became weaker, if not completely attenuated, after conditioning on the PrCa risk SNP (Supplementary Data 1). These are potentially due to (1) the previously identified associations of risk SNPs with PrCa at these loci may be mediated through the DNA methylation of these CpG sites identified in the current study, or (2) confounding effects (Supplementary Data 1). We estimated that the 15 CpG sites at novel loci and the 63 CpG sites independent of PrCa risk SNPs could explain 0.69% of familiar risk of PrCa (methods in Supplementary Information).

Based on annotation using ANNOVAR, there were substantial inflations of the “exonic” and “ncRNA exonic” regions for the identified PrCa-associated CpG sites when compared with the overall tested 77,243 CpG sites (chi-square tests: 15.28% versus 7.44%, *P* = 6.36 × 10^−16^; 5.53% versus 2.42%, *P* = 6.37 × 10^−8^) (Supplementary Table 2). Also, a substantial decreased proportion of the “intergenic” region was observed (chi-square test: 15.42% versus 25.10%, *P* = 1.13 × 10^−9^) (Supplementary Table 2).

Through an annotation of the 759 PrCa-associated CpG sites using eFORGE v1.2, there tends to be an overlap of their positions with regions containing lysine 4 mono-methylated H3 histone (H3K4me1) markers across 38 of 39 cell types included in the consolidated Roadmap Epigenomics Project, including blood tissues (Supplementary Fig. 2). This suggests that the identified CpG sites associated with PrCa risk may be enriched in enhancers and may be involved in transcriptional activation. We also observed significant enrichment for the associated CpG sites with positions of genes encoding transcription factors (*P* = 0.001).

For the identified 759 CpG sites showing an association in the PRACTICAL, CRUK, CAPS, BPC3, and PEGASUS consortia, we further evaluated their associations using independent UK Biobank data. In this analysis with far fewer PrCa cases, 554 CpG sites (73%) also showed an association at *P* < 0.05 with the same direction of effect (Supplementary Data 2). These suggested that the CpG-PrCa risk associations identified in the main analyses using data of the PRACTICAL, CRUK, CAPS, BPC3, and PEGASUS consortia were quite robust. We performed downstream analyses focusing on these 759 CpG sites.

### Potential target genes of the PrCa-associated CpG sites

Of the 759 PrCa-associated CpG sites, association analyses were performed for 689 pairs of CpG site-gene, including 613 CpG sites with 244 flanking genes. Overall, associations at a false discovery rate (FDR) < 0.05 were observed for methylation levels of 42 CpG sites with expression of 28 neighbor genes in blood tissue (Supplementary Table 3). Interestingly, we also observed several associations between DNA methylation and expression of genes encoding transcription factors at *P* < 0.05 (Supplementary Table 4). In the TCGA dataset of tumor-adjacent normal prostate tissue, albeit with a quite limited sample size (*n* = 34), we observed that 26 of the 37 associations that could be assessed showed the same direction of effect compared with that in the blood tissue (Supplementary Table 5). Among them, 11 showed statistical significance at *P* < 0.05 in this small dataset (Supplementary Table 5).

### Associations of potential target genes with PrCa risk

Of the 28 potential target genes of the identified CpG sites based on blood tissue analyses, blood tissue gene expression prediction models were built for 22 genes, and prostate tissue prediction models were built for 14 genes with a prediction performance (*R*^2^) of at least 0.01 (≥10% correlation). Using the S-PrediXcan method, we evaluated associations between the genetically predicted expression of these genes and PrCa risk. Of the 22 genes with blood tissue prediction models built, 18 demonstrated an association at FDR < 0.05 (Table 2). For 12 of them with prostate tissue prediction models built as well, nine showed an association at *P* < 0.05 (Table 2). For all of the nine genes except for *VPS53*, the direction of associations was consistent for the predicted expression in blood versus prostate tissue. Of two other genes with models built for prostate tissue only, *HLA-DOB* showed a significant association with PrCa risk (beta = 0.068, *P* = 2.65 × 10^−4^), and *C11orf21* did not show a significant association (*P* = 0.21).

**Table 2:** Associations between genetically predicted mRNA expression levels of candidate target genes of identified CpG sites and prostate cancer risk.

Gene	Blood tissue prediction model			Prostate tissue prediction model		
	*R*^2a^	OR (95% CI)^b^	*P* value^c^	*R*^2a^	OR (95% CI)^b^	*P* value^c^
*NCOA4*	0.14	3.80 (2.91–4.96)	1.39 × 10^−22^	0.18	1.41 (0.67–2.96)	0.36
*MDM4*	0.06	0.36 (0.29–0.45)	1.55 × 10^−19^	NA^d^	NA	NA
*BAIAP2L1*	0.03	2.21 (1.84–2.67)	5.86 × 10^−17^	NA	NA	NA
*GPR160*	0.46	0.78 (0.73–0.83)	2.03 × 10^−16^	NA	NA	NA
*PDK1*	0.09	1.86 (1.56–2.22)	8.81 × 10^−12^	NA	NA	NA
*TRIM26*	0.04	0.43 (0.34–0.55)	1.19 × 10^−11^	0.03	0.97 (0.53–1.78)	0.93
*UHRF1BP1*	0.40	1.11 (1.07–1.15)	1.99 × 10^−8^	0.21	1.18 (1.11–1.25)	3.24 × 10^−8^
*MCAT*	0.03	0.71 (0.62–0.80)	2.13 × 10^−8^	NA	NA	NA
*NUCKS1*	0.05	3.20 (2.12–4.83)	2.81 × 10^−8^	0.09	1.35 (1.17–1.55)	3.59 × 10^−5^
*C4B*	0.22	0.92 (0.89–0.95)	3.65 × 10^−8^	0.06	0.79 (0.69–0.89)	2.18 × 10^−4^
*PM20D1*	0.44	1.07 (1.04–1.10)	2.40 × 10^−7^	0.15	1.10 (1.06–1.14)	5.61 × 10^−7^
*CFAP44*	0.04	1.25 (1.14–1.36)	7.44 × 10^−7^	0.03	1.91 (1.61–2.26)	9.11 × 10^−14^
*LY6G5C*	0.48	1.06 (1.03–1.10)	9.52 × 10^−5^	0.17	1.11 (1.04–1.18)	1.16 × 10^−3^
*MICB*	0.37	0.94 (0.90–0.97)	8.86 × 10^−4^	0.18	0.89 (0.85–0.94)	3.32 × 10^−6^
*VAMP8*	0.01	0.66 (0.51–0.85)	1.37 × 10^−3^	0.09	1.08 (0.99–1.18)	0.08
*ZDHHC7*	0.10	0.80 (0.69–0.92)	2.52 × 10^−3^	0.15	0.83 (0.77–0.89)	3.78 × 10^−7^
*VAMP5*	0.10	1.19 (1.05–1.34)	5.01 × 10^−3^	NA	NA	NA
*VPS53*	0.63	1.03 (1.01–1.06)	9.02 × 10^−3^	0.45	0.95 (0.92–0.98)	2.86 × 10^−3^

^a^*R*^2^: mRNA expression prediction model performance (*R*^2^) derived using GTEx data.

^b^OR (odds ratio) and CI (confidence interval) per one standard deviation increase in genetically predicted mRNA expression levels.

^c^*P* value: derived from association analyses (two-sided); associations of genetically predicted expression in blood tissue with FDR < 0.05 are shown.

^d^NA: no prostate tissue prediction model was built.

### Associations showing consistent direction of effect

There were 25 CpG sites and 14 genes with consistent directions of association for the DNA methylation–gene expression–PrCa pathway (Table 3). For example, the CpG site cg20240347 located upstream of *MDM4*, and its DNA methylation level was positively associated with expression of *MDM4* (coefficient 0.21; *P* = 1.69 × 10^−14^). There was an inverse association between genetically predicted expression of *MDM4* and PrCa risk (OR = 0.36; *P* = 1.55 × 10^−19^). There was also evidence supporting the genetically predicted DNA methylation of cg20240347 to be associated with a decreased PrCa risk (OR = 0.93; *P* = 2.61 × 10^−19^). Interestingly, *MDM4* has been previously implicated as a potential target gene that is responsible for the identified association signal of index SNP rs4245739 in GWAS^25^, and in our recent TWAS study^27^. Our results highlight a possible role of the CpG site cg20240347 in the underlying biological mechanism of the link between *MDM4* and PrCa. Whether the DNA methylation of these CpG sites at the corresponding loci of the genes in Table 3 may play a role in PrCa etiology through the regulation of expression of these genes warrants further investigation. Ingenuity pathway analysis (IPA)^28^ suggested potential enrichment of cancer-related functions for the 14 implicated genes (Supplementary Table 6). The top canonical pathways identified included cell cycle (*P* = 0.033) and cancer drug resistance (*P* = 0.039). It is worth noting that based on the predicted DNA methylation–PrCa risk, DNA methylation–gene expression, and predicted gene expression–PrCa risk results, we also observed six CpG sites and four genes (*VAMP8*, *C4B*, *BAIAP2L1*, and *NCOA4*) with inconsistent directions of association for the DNA methylation–gene expression–PrCa pathway (Supplementary Table 7). Of these genes, *NCOA4*, *BAIAP2L1*, and *VAMP8* are candidate PrCa susceptibility genes identified in earlier TWAS^27,29,30^. Future work is needed to better understand these associations.

**Table 3:** Associations showing consistent direction of effect for the methylation–gene expression–prostate cancer risk pathway.

CpG site	Chr	Position	Associated gene	Classification	DNA methylation and prostate cancer risk		DNA methylation and gene expression		Gene expression and prostate cancer risk	
					OR	*P* value	Association coefficient	Association *P* value	OR	*P* value
cg20240347	1	204465584	*MDM4*	Upstream	0.93	2.61 × 10^−19^	0.21	1.69 × 10^−14^	0.36	1.55 × 10^−19^
cg15199181	1	205670604	*NUCKS1*	Upstream	0.94	5.10 × 10^−9^	−0.08	2.18 × 10^−3^	3.20	2.81 × 10^−8^
cg14893161	1	205819251	*PM20D1*	UTR5	0.97	1.11 × 10^−7^	−0.08	2.70 × 10^−3^	1.07	2.40 × 10^−7^
cg07167872	1	205819463		Upstream	0.97	1.47 × 10^−7^	−0.08	1.83 × 10^−3^		
cg24503407	1	205819492		Upstream	0.97	1.27 × 10^−7^	−0.08	2.78 × 10^−3^		
cg07157834	1	205819609		Upstream	0.96	1.07 × 10^−7^	−0.08	2.12 × 10^−3^		
cg02652597	2	85811292	*VAMP5*	Upstream	0.93	6.31 × 10^−7^	−0.16	8.76 × 10^−9^	1.19	5.01 × 10^−3^
cg10165864	2	173419899	*PDK1*	Upstream	0.89	6.02 × 10^−14^	−0.14	9.34 × 10^−8^	1.86	8.81 × 10^−12^
cg16797009	2	173472347		Downstream	0.90	2.31 × 10^−16^	−0.17	3.52 × 10^−10^		
cg25053018	2	173477995		Downstream	1.19	4.47 × 10^−20^	0.11	3.10 × 10^−5^		
cg07128416	3	113160490	*CFAP44*	Upstream	1.25	9.81 × 10^−11^	0.09	6.67 × 10^−4^	1.25	7.44 × 10^−7^
cg07054641	3	113160554		Upstream	1.22	6.46 × 10^−11^	0.09	6.47 × 10^−4^		
cg20138861	3	169775992	*GPR160*	Intronic	1.17	3.70 × 10^−14^	−0.11	5.97 × 10^−5^	0.78	2.03 × 10^−16^
cg24064041	6	30165027	*TRIM26*	Intronic	0.91	3.36 × 10^−9^	0.13	8.69 × 10^−7^	0.43	1.19 × 10^−11^
cg00266604	6	30178343		Intronic	1.21	2.05 × 10^−12^	−0.10	3.84 × 10^−4^		
cg12001709	6	31466798	*MICB*	Intronic	0.96	4.25 × 10^−8^	0.10	1.73 × 10^−4^	0.94	8.86 × 10^−4^
cg13892322	6	31648564	*LY6G5C*	Upstream	0.88	5.48 × 10^−7^	−0.12	4.42 × 10^−6^	1.06	9.52 × 10^−5^
cg22786465	6	31649502		Downstream	1.23	7.28 × 10^−10^	0.08	2.49 × 10^−3^		
cg02733847	6	31649519		Downstream	1.27	2.76 × 10^−7^	0.11	1.05 × 10^−4^		
cg25769566	6	31651278		Downstream	1.05	5.09 × 10^−8^	0.26	<2.00 × 10^−16^		
cg24520975	6	31651362		Downstream	1.15	6.87 × 10^−10^	0.10	2.37 × 10^−4^		
cg07306190	6	34760872	*UHRF1BP1*	Intronic	0.95	2.36 × 10^−8^	−0.33	<2.00 × 10^−16^	1.11	1.99 × 10^−8^
cg01715842	16	85045600	*ZDHHC7*	Upstream	1.05	2.95 × 10^−7^	−0.09	6.68 × 10^−4^	0.80	2.52 × 10^−3^
cg01799818	17	594735	*VPS53*	Intronic	1.10	7.40 × 10^−19^	0.09	4.81 × 10^−4^	1.03	9.02 × 10^−3^
cg10288850	22	43539588	*MCAT*	Upstream	2.18	6.23 × 10^−19^	−0.09	8.52 × 10^−4^	0.71	2.13 × 10^−8^

## Discussion

This is the first large-scale study to comprehensively evaluate associations of genetically predicted DNA methylation levels with PrCa risk. We identified 759 CpG sites whose predicted DNA methylation levels demonstrated an association after Bonferroni correction, including 15 located at novel loci. Of the 744 CpG sites located at known PrCa risk loci, 63 showed an association, even after conditioning on adjacent PrCa risk SNPs. In additional analyses involving gene expression, we observed some evidence suggesting that 25 CpG sites may influence PrCa risk via regulating expression of 14 candidate PrCa target genes. Our study provided substantial information to improve the understanding of genetics and etiology for PrCa, and it also generated multiple CpG sites as potential biomarkers for risk assessment of PrCa, the most common male malignancy globally.

For processing DNA methylation data for genetic model building, we performed quartile normalization for subjects followed by rank normalization for methylation levels, a standard approach widely used in the community for DNA methylation analyses^31^. We acknowledge, however, that such an approach could be suboptimal for CpG sites whose distributions of methylation do not resemble standard normal. Future endeavors for developing more sophisticated methods to deal with this are needed to pick up additional relevant signals. In this study, we identified 759 associated CpG sites, of which 42 were observed to be associated with expression of 28 flanking genes that were annotated by ANNOVAR, based on positions. For the other identified CpG sites, it is possible that genes that are not the most proximal ones could be target genes for local or distal regulation. However, to determine the exact target genes of these CpG sites involves additional lines of evidence besides statistical association, which is beyond the scope of this study. We observed 25 CpG sites with consistent directions of association for the DNA methylation–gene expression–PrCa pathway. Of the 14 linked genes, 10 (*MDM4*, *NUCKS1*, *PM20D1*, *VAMP5*, *GPR160*, *PDK1*, *UHRF1BP1*, *MCAT*, *LY6G5C*, and *VPS53*) demonstrated an association with PrCa risk in recent TWAS studies^27,30^. Furthermore, *MDM4* and *NUCKS1* have been previously implicated as potential target genes at GWAS-identified PrCa risk loci^25,32^. Our results incorporating DNA methylation provide additional insight into the potential mechanism for the link between these genes and PrCa development. Interestingly, in vitro experiments of silencing *PDK1* could decrease cell proliferation and inhibit the invasion and migration capability of PrCa cells^33^. Further functional studies are needed to better characterize whether there are potential regulatory effects of the identified 25 CpG sites on the expression of the 14 adjacent genes for PrCa development. Importantly, our design of integrating genome, methylome, and transcriptome data provides some evidence that 25 CpG sites may regulate expression of 14 candidate target genes, which further influences PrCa risk. Through the innovative integrative analyses harnessing large-scale human subject data, our study not only identifies several associations consistent with prior findings but it also uncovers potentially important roles of novel CpG sites and putative target genes (e.g., *CFAP44*, *TRIM26*, *MICB*, and *ZDHHC7*) in prostate tumorigenesis.

For the aim of identifying effective methylation biomarkers for risk assessment of PrCa, a design focusing on blood tissue would be optimal. Such a design could be suboptimal for characterizing the biological mechanism of PrCa development, when compared with the design using genetic instruments of DNA methylation levels identified in prostate tissue, considering potential tissue specificity in DNA methylation levels. On the other hand, research has shown that the genetic regulation of DNA methylation for many CpG sites tends to have a cross-tissue consistency, as indicated by studies comparing blood and different brain region tissues, and among lung, breast, and kidney tissues^20,34^. Furthermore, it is challenging to obtain prostate tissues from a large number of healthy individuals. Although prostate tumor-adjacent normal tissue methylation data are available in TCGA, tumor-adjacent normal tissue samples from PrCa patients may contain cancer cells; therefore, the methylation profile of these samples could be different from that of normal prostate tissue samples from healthy men. The statistical power for the model building using TCGA data could also be low due to the relatively small sample size available. In this study, for assessing DNA methylation–gene expression associations to determine potential target genes of identified CpG sites, besides using data from blood tissue (Supplementary Table 3), we also leveraged data from tumor-adjacent normal prostate tissue in TCGA. Despite a small sample size, we observed evidence supporting many of the associations identified using blood tissue data (Supplementary Table 5). For evaluating predicted gene expression–PrCa risk associations, our analyses using prostate tissue gene expression prediction models also support many of the associations identified using blood tissue prediction models (Table 2).

In the current work, a large number of subjects (*N* = 1595) in the reference FHS dataset was used for the DNA methylation prediction model building. Aligned with the huge sample size for our main association analyses for PrCa risk (79,194 cases and 61,112 controls), our study provides an unparalleled opportunity to detect the DNA methylation–PrCa associations. The use of genetic instruments rendered our study as potentially less susceptible to several limitations commonly encountered in conventional epidemiological studies, such as selection bias and reverse causation. On the other hand, it is worth noting that similar to TWAS, the associations observed in our analyses focusing on CpG sites are also vulnerable to confounding due to pleiotropy and co-localization of genetic signals. For instance, it would be difficult to distinguish a situation in which one causal methylation quantitative trait locus (mQTL) regulates the methylation of two CpG sites from a scenario in which two CpG sites have two causal mQTLs that are in linkage disequilibrium (LD) with each other. Correlated total methylation levels across CpG sites, correlated predicted DNA methylation across CpG sites, as well as shared genetic variants between DNA methylation genetic prediction models and gene expression prediction models, could all lead to spurious associations in our analyses^35^. When faced with two correlated predictors, regularized regression models like elastic net will randomly down weight one of them, which may be the true causal variant. Despite these potential limitations, our study generated a list of promising PrCa-associated CpG sites that warrant further investigation. By integrating the relationship between DNA methylation, gene expression, and PrCa risk using multi-omics data from different sources, we were able to identify consistent associations of the DNA methylation–gene expression–PrCa risk pathway. This supports a very interesting hypothesis that methylation at selected CpG sites could influence PrCa risk through the regulation of expression of adjacent target genes, which warrants further investigation. The current work generates a list of promising CpG sites showing an association with PrCa, which can be investigated further in future studies that directly measure levels of these CpG sites. Identification of circulating DNA methylation biomarkers could be useful for PrCa risk assessment.

In conclusion, in a large-scale study to evaluate associations between genetically predicted DNA methylation levels and PrCa risk, we identified 759 CpG sites that showed an association, including 15 at novel loci, and an additional 63 that represent association signals independent of known risk variants. We also observed that specific CpG sites may influence PrCa risk via regulating expression of candidate PrCa target genes. Further investigation of these findings will provide additional insight into the biology and genetics of PrCa, as well as facilitate risk assessment of PrCa.

## Methods

### Study design

**Fig. 2:**
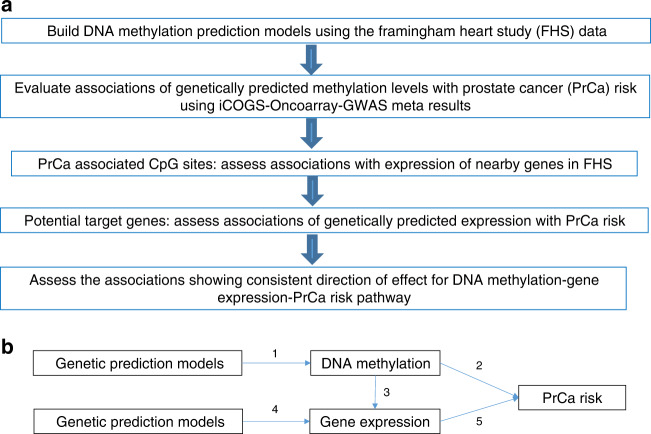
Study design. **a** Study design flow chart; **b** overview of the integrative-omics analysis. (1) Genetic prediction model building for blood DNA methylation levels; (2) associations of genetically predicted DNA methylation in blood and prostate cancer risk; (3) expression quantitative trait methylation; (4) genetic prediction models for blood and prostate tissue gene expression levels; (5) associations of genetically predicted gene expression in blood and prostate tissue with prostate cancer risk. Results in 1 were based on data of the Framingham Heart Study (FHS) (*N* = 1595). Results in 2 and 5 were based on the summary statistics of the PRACTICAL, CRUK, CAPS, BPC3, and PEGASUS consortia (*N* = 79,194 cases and 61,112 controls). Results in 3 were based on data of the FHS (*N* = 1367) and The Cancer Genome Atlas (*N* = 34). Results in 4 were based on data of the Genotype-Tissue Expression project (version 8).

The overall study design is shown in Fig. 2. First, we built comprehensive genetic prediction models for DNA methylation levels by using data of the Framingham Heart Study (FHS). After external validation, we selected methylation models with satisfactory prediction performance for association analyses of genetically predicted methylation levels with PrCa risk, by using data of the PRACTICAL consortia which involves 79,194 cases and 61,112 controls. For CpG sites showing an association with PrCa risk, we assessed associations of their methylation with expression of adjacent genes (FHS, *N* = 1367), to identify potential target genes of these CpG sites. For the suggested candidate target genes, we further assessed associations of their genetically predicted expression with PrCa risk.

### Building of DNA methylation prediction models

We obtained the individual level genome-wide genotyping and white blood cell DNA methylation data from the FHS Offspring Cohort (dbGaP accession numbers: phs000342 and phs000724). The details of the FHS Offspring Cohort have been described elsewhere^36^. In brief, DNA was genotyped using the Affymetrix 500 K array, and DNA methylation was profiled using the Illumina HumanMethylation450 BeadChip. The genotype data were imputed to the Haplotype Reference Consortium reference panel^37^. SNPs with high imputation quality (*R*^2^ ≥ 0.8), minor allele frequency ≥0.05, included in the HapMap Phase 2 version, and those that were not strand ambiguous were used to build DNA methylation prediction models. For DNA methylation data, the “minfi” package^38^ was used to filter out low-quality samples, exclude low-quality methylation probes, estimate cell-type composition, and calculate methylation beta values. We performed quantile normalization to bring the methylation profile of each sample to the same scale, and rank normalization for each CpG site to map each set of DNA methylation values to a standard normal. We adjusted for age, sex, six cell-type composition variables, and the top ten principal components (PCs) derived from genotype data. Genetic and DNA methylation data from 1595 genetically unrelated subjects of European descent were used to build DNA methylation prediction models for this study.

For each CpG site, we built a genetic model to predict DNA methylation levels using the elastic net method as implemented in the “glmnet” package of R, with *α* = 0.5^39–41^ (Supplementary Software 1). Genetic variants flanking a 2-Mb window of each CpG site were used to build the model. Tenfold cross-validation was used for internal validation. Prediction *R*^2^ values, the square of the correlation between predicted and measured methylation levels, were used to estimate the model prediction performance.

### External validation of the models

To further evaluate the validity of the built methylation prediction models, we performed external validation using data from 883 unrelated healthy female participants of European descent included in The Women’s Health Initiative (WHI) (dbGaP accession numbers: phs000315, phs000675, and phs001335). Genotype data and white blood cell DNA methylation data were processed using a similar approach, as described above. The predicted DNA methylation for each CpG site was calculated using the models that were established using FHS data, and then compared with the measured level using Spearman’s correlation.

### Associations between predicted methylation and PrCa

Considering that our model external validation dataset WHI included females only, and that there is a high concordance of the model performance (*R*^2^) in FHS and WHI, we included DNA methylation prediction models (1) with a *R*^2^ ≥ 0.01 (≥10% correlation between predicted and measured methylation levels) in FHS, a standard criterion used in TWAS for gene expression^27,39,42–44^, heritability of which tends to be similar to that of DNA methylation in blood^31,45^, and (2) for probes with no SNPs within the probe-binding site, considering that the measurement of DNA methylation levels for such probes tends to be unbiased^46^. Overall, we evaluated associations between genetically predicted methylation levels of 77,243 CpG sites with PrCa risk.

We estimated the association between genetically predicted DNA methylation levels and PrCa risk using S-PrediXcan, which has been described elsewhere^47^ (Supplementary Software 1). We used the summary statistics data for the association of genetic variants with PrCa risk that had been generated from 79,194 PrCa cases and 61,112 controls of European ancestry in the PRACTICAL, CRUK, CAPS, BPC3, and PEGASUS consortia^26,48^. In brief, 46,939 PrCa cases and 27,910 controls were genotyped using OncoArray, which included 570,000 SNPs (http://epi.grants.cancer.gov/oncoarray/). Also included were data from several previous PrCa GWAS of European ancestry: UK stage 1 and stage 2, CaPS 1 and CaPS 2, BPC3, NCI PEGASUS, and iCOGS. These genotype data were imputed using the June 2014 release of the 1000 Genomes Project data as reference. Logistic regression summary statistics were then meta-analyzed using an inverse variance fixed effect approach.

A Bonferroni-corrected threshold of *P* < 6.47 × 10^−7^ (0.05/77,243) was used to determine a statistically significant association. For CpG sites showing a significant association between genetically predicted methylation levels with PrCa risk, we further evaluated whether the observed associations were independent of nearby PrCa risk variants identified in GWAS or fine-mapping studies, by performing GCTA-COJO analysis^49^. For this analysis, the risk SNP showing the most significant association with PrCa risk in the PRACTICAL, CRUK, CAPS, BPC3, and PEGASUS consortia was adjusted for calculating association betas and standard errors of DNA methylation predicting SNPs with PrCa risk. These association statistics were then used for re-running the S-PrediXcan analyses.

### Familial relative risk of PrCa explained by novel CpG sites

For PrCa-associated CpG sites that were located at novel loci or independent from known PrCa risk variants, we used the linkage disequilibrium (LD) score regression method^50^ to evaluate the proportion of familial relative risk of PrCa that could be explained by predicted methylation levels of these CpG sites. In brief, we firstly applied the prediction models of these CpGs to the genetic data of male controls included in the pancreatic cancer GWAS data (*N* = 3655) to generate the predicted methylation of these CpGs for each of the participants. Detailed information for this dataset, quality control, and imputation has been described elsewhere^51^. We further used the formula *Z*^2^ = 1 + (*N*_*T*_*l*/*M*)/$h$^2^ to estimate the heritability explained by these CpG sites. Here for each CpG, *Z* represents the *Z* score of the association between the predicted methylation and PrCa risk; *N*_*T*_ represents the number of individuals included in the GWAS of the PRACTICAL, CRUK, CAPS, BPC3, and PEGASUS consortia, namely, 140,306; *l* represents the LD score of the CpG of interest; *M* represents the number of CpG sites that were significantly associated with PrCa risk; and $h$^2^ is the estimated heritability of PrCa risk that could be explained by the predicted methylation of the CpG sites of interest. The LD score for each CpG was estimated by adding up the squared Pearson correlation coefficient (*R*^2^) of the CpG of interest with all the other CpG sites. Finally, after fitting a linear regression model using data of all these CpGs, the estimated heritability of PrCa risk that could be explained by the predicted methylation of the CpGs of interest, along with the standard error and *P* value, were estimated. Given that the heritability of PrCa was estimated to be 57%^52^, the familial relative risk of PrCa that could be explained by predicted methylation levels of these CpGs was calculated as ℎ^2^/0.57.

### Validation of identified CpG sites using the UK Biobank

Individual level data of the UK Biobank were used to validate the identified associated CpG sites. The UK Biobank released GWAS data on ~500,000 individuals^53^. PrCa cases were determined by combining Hospital Episode Statistics (HES) data and self-reported data. Specifically, cases were defined as hospital admission, type of cancer, or cause of death due to ICD-9 185.9 or ICD-10 C61 or a self-reported cancer code. We calculated associations of genetically predicted DNA methylation of the identified CpG sites with PrCa risk, adjusting for age, age^2^, and top 20 PCs provided by the UK Biobank. As the number of cases in the UK Biobank is substantially smaller than that in the PRACTICAL, CRUK, CAPS, BPC3, and PEGASUS consortia, we used results from the UK Biobank to confirm the validity of the CpG sites identified in analyses of the consortia data, instead of using their results to filter out CpG sites.

### Functional annotation of PrCa-associated CpG sites

We annotated the position and genomic region information of the identified PrCa-associated CpG sites through ANNOVAR^54^. The CpG sites were annotated into one of 13 functional categories, including exonic, intronic, intergenic, upstream, 3′-UTR, 5′-UTR, ncRNA intronic, ncRNA exonic, splicing, downstream, upstream/downstream, 5′-UTR/3′-UTR, and exonic/splicing. We used eFORGE^55^ v1.2 to assess whether the identified CpG sites were enriched in DNase I hypersensitive sites (DHSs) and loci overlapping with various histone modification types, such as H3K27me3, H3K36me3, H3K4me3, H3K9me3, and H3K4me1, across different tissues and cell lines available in the Roadmap Epigenomics Project^56^, the Encyclopedia of DNA Elements (ENCODE)^57^ and the BLUPRINT Epigenome^58^. For each CpG site set of interest, eFORGE performs an overlap analysis against the functional elements for each tissue or cell line separately, and then counts the number of overlaps. A background distribution of the expected overlap counts for the CpG site set of interest is obtained by picking sets of CpG sites with the same number as the test set, matched for gene relationship and CpG island relationship annotation. The matched background sets are then overlapped with the functional elements and the background distribution of overlaps are determined. 1000 matched sets are used. The enrichment value for the test set is expressed as the -log_10_(binomial *P* value). Enrichments outside the nominal 95th and 99th percentile of the binomial distribution (after Benjamini–Yekutieli multiple testing correction) are considered significant. We also evaluated whether the associated CpG sites were enriched in loci of genes encoding transcription factors^59^.

### Determine genes associated with identified CpG sites

For CpG sites with genetically predicted DNA methylation levels significantly associated with PrCa risk, we evaluated associations between methylation and expression levels of genes flanking their loci by using data from the FHS Offspring Cohort (dbGaP accession numbers: phs000363 and phs000724) and The Cancer Genome Atlas (TCGA). Details of the FHS Offspring Cohort, DNA methylation, and gene expression data have been described elsewhere^36,60,61^. Overall, DNA methylation and gene expression data were available for 1367 unrelated individuals. For the CpG sites showing a significant association with PrCa risk, associations between the normalized methylation levels in beta values and normalized expression levels of genes flanking the CpG sites were estimated, after adjusting for age, sex, top PCs, and estimated cell-type compositions based on methylation data. We further assessed significant methylation–gene expression associations identified in blood tissue analyses in adjacent normal prostate tissue of PrCa patients in the TCGA (*N* = 34). The processing of DNA methylation and gene expression data has been described elsewhere^62,63^.

### Associations of potential target genes with PrCa risk

For genes whose expression levels were associated with DNA methylation levels, we assessed whether the genetically predicted expression levels of these genes in blood and prostate tissue were also associated with PrCa risk^44,64,65^. We used prediction models developed using the PrediXcan method (Elastic Net) and leveraging data from the v8 version of the Genotype-Tissue Expression dataset (GTEx) project (http://predictdb.org/). Details of the methods of building gene expression prediction models using SNPs have been described elsewhere^44,47,66^. The prediction models were used to estimate the associations between genetically predicted gene expression levels and PrCa risk in the PRACTICAL, CRUK, CAPS, BPC3, and PEGASUS consortia using S-PrediXcan^47^.

### Associations showing a consistent direction of effect

We assessed the associations between genetically predicted DNA methylation levels and PrCa risk, associations between DNA methylation and gene expression levels, and the associations between genetically predicted gene expression and PrCa risk to assess associations showing consistent direction of effect for the DNA methylation–gene expression–PrCa risk pathway. This could indicate the possibility that genetically predicted DNA methylation might putatively influence PrCa risk through the regulation of expression of flanking target genes.

### Functional enrichment analysis

We performed functional enrichment analysis for the identified genes consistent with the DNA methylation–gene expression–PrCa risk pathway. Canonical pathways, top associated diseases and biofunctions, and top networks associated with these genes were estimated using IPA software^28^.

### Reporting summary

Further information on research design is available in the Nature Research Reporting Summary linked to this article.

## Supplementary information

Supplementary Information

Description of Additional Supplementary Files

Supplementary Data 1

Supplementary Data 2

Supplementary Software 1

Reporting Summary

## Data Availability

The OncoArray genotype data and relevant covariate information (i.e., ethnicity, country, principal components, etc.) for prostate cancer study are available in dbGAP (Accession no.: phs001391.v1.p1). In total, 47 of the 52 OncoArray studies, encompassing ~90% of the individual samples, are available. The previous meta-analysis summary results and genotype data are currently available in dbGaP (Accession no.: phs001081.v1.p1). The datasets of FHS Offspring Cohort and WHI are publicly available via dbGaP (www.ncbi.nlm.nih.gov/gap): dbGaP Study Accession: phs000342 and phs000724 for FHS, and phs000315, phs000675, and phs001335 for WHI. TCGA data can be accessed through the Genomic Data Commons Data Portal.

## Code availability

The relevant codes are available in the Supplementary Software 1.
