# Supplementary material for: An integrative multi-omics analysis to identify candidate DNA methylation biomarkers related to prostate cancer risk
Source: Nat Commun. 2020 Aug 6;11:3905. doi: 10.1038/s41467-020-17673-9 (PMC7413371; doi:10.1038/s41467-020-17673-9)
Supplement: Supplementary file 1 — Supplementary Information [file 41467_2020_17673_MOESM1_ESM.pdf]

An integrative multi-omics analysis to identify candidate DNA methylation biomarkers related to prostate cancer risk

Wu et al

Supplementary Table 1. Sixty-three methylation-prostate cancer associations independent from prostate cancer risk variants[a] for CpG sites at prostate cancer risk loci

CpG site	Chr	Position (build37)	Classification	R^{2}[b]	OR (95% CI)[c]	*P* value[d]	risk SNP	Distance to the risk SNP (kb)	*P* value after adjusting for risk SNP[e]
cg14454477	2	43903900	intronic	0.10	0.89 (0.85-0.93)	1.85×10^{-8}	rs1465618	350.0	2.61×10^{-7}
cg10804687	6	29859520	downstream	0.05	1.22 (1.14-1.30)	5.62×10^{-9}	rs7767188	214.3	5.45×10^{-8}
cg19872019	6	29980960	ncRNA_exonic	0.06	0.82 (0.78-0.87)	6.37×10^{-12}	rs7767188	92.8	5.14×10^{-7}
cg03553308	6	30069250	intergenic	0.22	0.91 (0.89-0.93)	3.52×10^{-14}	rs7767188	4.5	3.19×10^{-8}
cg24064041	6	30165027	intronic	0.18	0.91 (0.89-0.94)	3.36×10^{-9}	rs12665339	436.2	2.10×10^{-7}
cg02541301	6	30166173	intronic	0.02	0.73 (0.67-0.80)	7.44×10^{-11}	rs12665339	435.1	2.54×10^{-8}
cg09609649	6	30458060	exonic	0.02	0.75 (0.69-0.82)	3.55×10^{-10}	rs12665339	143.2	4.49×10^{-8}
cg19109457	6	30460484	intronic	0.23	1.07 (1.04-1.10)	1.56×10^{-7}	rs12665339	140.7	6.09×10^{-7}
cg08743794	6	30656577	exonic	0.01	0.55 (0.48-0.63)	2.71×10^{-18}	rs12665339	55.3	8.57×10^{-17}
cg26004235	6	30656582	exonic	0.01	0.72 (0.66-0.79)	7.71×10^{-12}	rs12665339	55.4	9.51×10^{-8}
cg06206827	6	30709045	exonic	0.05	0.84 (0.80-0.88)	5.80×10^{-13}	rs12665339	107.8	1.33×10^{-8}
cg17067528	6	30712517	upstream	0.01	0.79 (0.73-0.86)	8.02×10^{-9}	rs12665339	111.3	3.98×10^{-7}
cg08951271	6	30850543	UTR5	0.06	0.80 (0.75-0.85)	1.64×10^{-12}	rs2596546	478.8	2.51×10^{-7}
cg12433575	6	30881464	intronic	0.02	0.72 (0.66-0.78)	6.48×10^{-15}	rs2596546	447.9	3.19×10^{-12}
cg26467571	6	30882355	intronic	0.03	0.76 (0.70-0.82)	4.03×10^{-12}	rs2596546	447.0	6.07×10^{-7}
cg16958594	6	30882708	exonic	0.69	0.96 (0.95-0.97)	5.08×10^{-9}	rs2596546	446.7	7.86×10^{-8}
cg15978899	6	30882994	exonic	0.59	0.96 (0.94-0.97)	2.38×10^{-9}	rs2596546	446.4	5.85×10^{-9}
cg00933603	6	30883001	exonic	0.61	0.96 (0.94-0.97)	1.52×10^{-8}	rs2596546	446.4	4.06×10^{-8}
cg10158679	6	30883074	intronic	0.61	0.96 (0.94-0.97)	5.48×10^{-9}	rs2596546	446.3	2.71×10^{-8}
cg00244776	6	30883192	exonic	0.51	0.95 (0.94-0.97)	2.13×10^{-8}	rs2596546	446.2	5.29×10^{-8}
cg02149965	6	30883203	exonic	0.55	0.95 (0.94-0.97)	5.94×10^{-10}	rs2596546	446.2	8.70×10^{-9}
cg08827454	6	30922981	upstream/downstream	0.06	0.84 (0.80-0.89)	1.25×10^{-10}	rs2596546	406.4	5.69×10^{-7}
cg03059420	6	30923241	upstream	0.02	0.70 (0.64-0.77)	2.02×10^{-13}	rs2596546	406.1	5.16×10^{-12}
cg11935153	6	30923306	upstream	0.06	0.86 (0.82-0.91)	4.61×10^{-8}	rs2596546	406.1	2.21×10^{-8}
cg15878568	6	30923865	intergenic	0.21	0.92 (0.90-0.95)	7.14×10^{-9}	rs2596546	405.5	1.93×10^{-8}
cg11934771	6	31021796	intronic	0.08	1.24 (1.18-1.30)	5.19×10^{-19}	rs2596546	307.6	1.64×10^{-8}
cg08961072	6	31591771	intronic	0.01	0.69 (0.62-0.77)	1.51×10^{-12}	rs2596546	262.4	1.24×10^{-7}
cg16220567	6	31631762	exonic	0.06	1.15 (1.10-1.20)	8.98×10^{-9}	rs2596546	302.4	1.33×10^{-7}
cg06670599	6	31631801	exonic	0.05	1.16 (1.10-1.22)	1.66×10^{-7}	rs2596546	302.4	4.61×10^{-7}
cg22786465	6	31649502	intergenic	0.04	1.23 (1.15-1.31)	7.28×10^{-10}	rs2596546	320.1	9.86×10^{-9}
cg22708150	6	31649619	intergenic	0.04	1.18 (1.12-1.24)	1.31×10^{-9}	rs2596546	320.2	1.43×10^{-8}

cg21036162	6	31649728	intergenic	0.03	1.28 (1.19-1.38)	1.39×10^{-10}	rs2596546	320.3	5.18×10^{-8}
cg24520975	6	31651362	intergenic	0.10	1.15 (1.10-1.20)	6.87×10^{-10}	rs2596546	322.0	4.49×10^{-8}
cg17391620	6	31734471	exonic	0.07	1.17 (1.12-1.23)	2.19×10^{-11}	rs2596546	405.1	4.05×10^{-7}
cg26472225	6	31832238	intronic	0.03	1.37 (1.25-1.50)	5.95×10^{-11}	rs3096702	360.1	3.74×10^{-7}
cg10917426	6	31867698	UTR3	0.04	0.78 (0.73-0.83)	1.19×10^{-13}	rs3096702	324.6	1.24×10^{-8}
cg08975528	6	31867700	UTR3	0.02	0.69 (0.63-0.76)	1.85×10^{-14}	rs3096702	324.6	2.31×10^{-10}
cg07180897	6	32729130	intronic	0.64	1.04 (1.03-1.06)	3.17×10^{-8}	rs3129859	328.2	3.98×10^{-7}
cg00755130	6	32729587	exonic	0.21	0.91 (0.89-0.93)	4.53×10^{-15}	rs3129859	328.6	2.17×10^{-7}
cg27160348	6	32729590	exonic	0.21	0.89 (0.87-0.92)	1.53×10^{-17}	rs3129859	328.7	1.05×10^{-7}
cg25736982	6	160182554	ncRNA_exonic	0.21	0.92 (0.90-0.95)	4.80×10^{-9}	rs651164	398.8	1.21×10^{-8}
cg23829577	6	160183769	exonic	0.04	0.80 (0.74-0.86)	4.70×10^{-9}	rs651164	397.6	1.21×10^{-7}
cg21110739	6	160768369	intergenic	0.02	2.03 (1.73-2.39)	1.12×10^{-17}	rs4646284	186.8	9.37×10^{-10}
cg25313204	6	160768801	upstream	0.08	1.17 (1.13-1.22)	7.46×10^{-17}	rs4646284	187.3	3.23×10^{-11}
cg23898998	6	160782998	intronic	0.08	1.35 (1.27-1.43)	2.61×10^{-22}	rs4646284	201.5	1.73×10^{-11}
cg14550828	6	160876992	intergenic	0.02	1.61 (1.47-1.77)	6.18×10^{-23}	rs4646284	295.4	2.23×10^{-13}
cg12196573	7	27195602	intronic	0.01	1.30 (1.18-1.44)	1.97×10^{-7}	rs200362064	395.6	1.70×10^{-7}
cg02643054	7	27206544	ncRNA_intronic	0.02	1.31 (1.20-1.42)	2.07×10^{-10}	rs200362064	384.7	7.77×10^{-9}
cg06795527	7	27245018	ncRNA_exonic	0.10	0.87 (0.84-0.91)	2.47×10^{-11}	rs200362064	346.2	3.75×10^{-9}
cg16196175	7	27289120	intergenic	0.02	0.74 (0.67-0.81)	3.19×10^{-10}	rs200362064	302.1	3.15×10^{-8}
cg06521347	8	128139451	intergenic	0.09	0.79 (0.75-0.82)	1.17×10^{-27}	rs11986220	392.2	1.11×10^{-27}
cg23203918	8	128235836	intergenic	0.05	1.30 (1.23-1.38)	5.59×10^{-20}	rs11986220	295.9	1.25×10^{-24}
cg17095489	8	128264282	ncRNA_intronic	0.03	0.69 (0.64-0.75)	2.16×10^{-21}	rs11986220	267.4	1.97×10^{-33}
cg15704662	8	128388831	ncRNA_intronic	0.07	0.82 (0.78-0.87)	8.12×10^{-13}	rs10505477	18.6	2.61×10^{-18}
cg14289643	8	128428869	exonic	0.02	0.47 (0.43-0.51)	1.70×10^{-57}	rs6983267	15.6	3.05×10^{-8}
cg14036981	11	68920648	ncRNA_intronic	0.24	1.07 (1.05-1.10)	3.04×10^{-8}	rs12275055	60.7	1.49×10^{-7}
cg25179853	11	68924577	ncRNA_intronic	0.12	1.20 (1.16-1.25)	7.56×10^{-24}	rs12275055	56.8	2.32×10^{-10}
cg23740940	11	68924746	ncRNA_intronic	0.32	1.10 (1.08-1.12)	1.37×10^{-20}	rs12275055	56.6	1.69×10^{-12}
cg07882059	11	68924751	ncRNA_intronic	0.34	1.09 (1.07-1.11)	1.17×10^{-16}	rs12275055	56.6	4.52×10^{-11}
cg03469862	11	68924853	ncRNA_intronic	0.28	1.11 (1.08-1.13)	4.16×10^{-21}	rs12275055	56.5	4.40×10^{-14}
cg26453588	22	43506021	upstream	0.21	1.29 (1.25-1.32)	3.66×10^{-67}	rs5759167	5.8	4.14×10^{-18}
cg04042468	22	43506033	upstream	0.15	1.34 (1.30-1.39)	2.10×10^{-70}	rs5759167	5.8	3.64×10^{-15}
cg20550677	22	43506316	upstream	0.07	1.68 (1.59-1.78)	4.23×10^{-72}	rs5759167	6.1	3.44×10^{-13}

[a] Risk SNPs identified in previous GWAS or fine-mapping studies.

[b] R^2: model prediction performance (R^2) derived using FHS data.

[c] OR (odds ratio) and CI (confidence interval) per one standard deviation increase in genetically predicted DNA methylation

[d] *P* value: derived from association analyses of 79,194 cases and 61,112 controls (two-sided); associations with $p \leq 6.47 \times 10^{-7}$ based on Bonferroni correction of 77,243 tests (0.05/77,243) were shown;

[e] using COJO method

Supplementary Table 2. Genomic annotation of prostate cancer associated CpG site locations. Categories were annotated through ANNOVAR. Substantial inflations of “exonic” and “ncRNA_exonic”, and substantial decreased proportion of “intergenic” are found for prostate cancer associated CpG sites compared with the overall tested 77,243 CpG sites.

**Classification**	**Identified CpG sites associated with prostate cancer risk (n=759)**	**Overall tested CpG sites (N=77,243)**	***P* for difference**
intronic	268 (35.31%)	28053 (36.32%)	0.59
intergenic	117 (15.42%)	19388 (25.10%)	1.13×10^{-9}
upstream	87 (11.46%)	10912 (14.13%)	0.04
exonic	116 (15.28%)	5749 (7.44%)	6.36×10^{-16}
ncRNA_intronic	52 (6.85%)	4184 (5.42%)	0.10
5’-UTR	28 (3.69%)	3075 (3.98%)	0.75
3’-UTR	35 (4.61%)	2765 (3.58%)	0.15
ncRNA_exonic	42 (5.53%)	1868 (2.42%)	6.37×10^{-8}
downstream	8 (1.05%)	795 (1.03%)	1.00
upstream;downstream	6 (0.79%)	426 (0.55%)	0.52
splicing	0 (0.00%)	17 (0.02%)	1.00
5’-UTR; 3’-UTR	0 (0.00%)	8 (0.01%)	1.00
exonic;splicing	0 (0.00%)	1 (0.001%)	1.00
ncRNA_splicing	0 (0.00%)	2 (0.003%)	1.00

chi-square tests (two-sided)

Supplementary Table 3. Associations between methylation levels of prostate cancer associated CpG sites and expression of annotated adjacent genes in white blood cells in the Framingham Heart Study*

CpG site	Adjacent gene	Classification	Association beta	Association *P* value[a]
cg01799818	*VPS53*	intronic	0.09	4.81×10^{-4}
cg13731761	*C11orf21*	exonic	-0.21	2.20×10^{-14}
cg26598899	*C11orf21*	exonic	-0.18	1.03×10^{-11}
cg21162977	*RRAGA*	exonic	-0.09	8.82×10^{-4}
cg26751972	*HLA-F*	exonic	-0.15	1.43×10^{-8}
cg24064041	*TRIM26*	intronic	0.13	8.69×10^{-7}
cg00266604	*TRIM26*	intronic	-0.10	3.84×10^{-4}
cg12001709	*MICB*	intronic	0.10	1.73×10^{-4}
cg10970124	*CSNK2B*	UTR5	-0.10	2.88×10^{-4}
cg13892322	*LY6G5C*	upstream	-0.12	4.42×10^{-6}
cg22786465	*LY6G5C*	downstream	0.08	2.49×10^{-3}
cg02733847	*LY6G5C*	downstream	0.11	1.05×10^{-4}
cg25769566	*LY6G5C*	downstream	0.26	$<2.00 \times 10^{-16}$
cg24520975	*LY6G5C*	downstream	0.10	2.37×10^{-4}
cg13197078	*C4B*	intronic	-0.13	3.24×10^{-6}
cg11239749	*HLA-DOB*	intronic	0.20	3.81×10^{-14}
cg19350197	*HLA-DOB*	exonic	0.24	$<2.00 \times 10^{-16}$
cg25824217	*HLA-DPA1*	intronic	0.16	2.69×10^{-9}
cg07306190	*UHRF1BP1*	intronic	-0.33	$< 2.00 \times 10^{-16}$
cg10288850	*MCAT*	upstream	-0.09	8.52×10^{-4}
cg06298701	*NCOA4*	intronic	-0.08	1.90×10^{-3}
cg17620335	*NCOA4*	intronic	-0.08	2.51×10^{-3}
cg01330312	*NCOA4*	intronic	-0.12	1.28×10^{-5}
cg07185131	*EHBP1*	upstream	-0.08	2.61×10^{-3}
cg01715842	*ZDHHC7*	upstream	-0.09	6.68×10^{-4}
cg20056908	*VAMP8*	UTR3	0.20	3.03×10^{-14}
cg02652597	*VAMP5*	upstream	-0.16	8.76×10^{-9}
cg15059474	*BAIAP2L1*	intronic	0.11	9.72×10^{-5}
cg08336300	*SESN1*	intronic	-0.11	2.34×10^{-5}
cg17117243	*SESN1*	intronic	-0.15	1.87×10^{-8}

cg07128416	*CFAP44*	upstream	0.09	6.67×10^{-4}
cg07054641	*CFAP44*	upstream	0.09	6.47×10^{-4}
cg20138861	*GPR160*	intronic	-0.11	5.97×10^{-5}
cg10165864	*PDK1*	upstream	-0.14	9.34×10^{-8}
cg16797009	*PDK1*	downstream	-0.17	3.52×10^{-10}
cg25053018	*PDK1*	downstream	0.11	3.10×10^{-5}
cg20240347	*PIK3C2B*	downstream	0.11	2.59×10^{-5}
cg20240347	*MDM4*	upstream	0.21	1.69×10^{-14}
cg15199181	*NUCKS1*	upstream	-0.08	2.18×10^{-3}
cg14893161	*PM20D1*	UTR5	-0.08	2.70×10^{-3}
cg07167872	*PM20D1*	upstream	-0.08	1.83×10^{-3}
cg24503407	*PM20D1*	upstream	-0.08	2.78×10^{-3}
cg07157834	*PM20D1*	upstream	-0.08	2.12×10^{-3}

* Linear regression analyses (two-sided) adjusted for covariates of age, sex, top PCs and estimated cell type compositions

[a] *P* value: associations with *fdr*<0.05 were shown

Supplementary Table 4. Associations between methylation levels of prostate cancer associated CpG sites and expression of genes encoding transcription factors at *P*<0.05 in white blood cells in the Framingham Heart Study

CpG site	Adjacent gene	Family	Protein	Classification	Association beta	Association *P* value[a]
cg10917426	*ZBTB12*	ZBTB	ENSP00000364677	UTR3	0.06	0.04
cg19376664				UTR3	-0.08	0.005
cg14538532				UTR3	-0.06	0.02
cg06431527	*PBX2*	Homeobox	ENSP00000364190	upstream	0.05	0.05
cg24038745	*POU5F1*	Pou	ENSP00000495779;ENSP00000419298;	exonic	-0.05	0.05
cg26416811	*RFX6*	RFX	ENSP00000332208;	upstream	-0.07	0.009
cg03510041	*FOXP4*	Fork_head	ENSP00000362154;ENSP00000386958;ENSP00000309823; ENSP00000362151;ENSP00000362148;	intronic	-0.07	0.01

* Linear regression analyses (two-sided) adjusted for covariates of age, sex, top PCs and estimated cell type compositions

Supplementary Table 5. Associations between methylation levels of prostate cancer associated CpG sites and expression of annotated adjacent genes in tumor adjacent normal prostate tissue samples in The Cancer Genome Atlas

CpG site	Adjacent gene	Classification	Association beta	Association *P* value[a]
cg01799818	*VPS53*	intronic	-0.17	0.32
**cg13731761***	***C11orf21***	exonic	-0.35	0.04
**cg26598899***	***C11orf21***	exonic	-0.57	0.0004
cg21162977*	*RRAGA*	exonic	-0.13	0.45
cg26751972*	*HLA-F*	exonic	-0.28	0.11
cg24064041	*TRIM26*	intronic	-0.06	0.73
cg12001709*	*MICB*	intronic	0.29	0.10
cg10970124*	*CSNK2B*	UTR5	-0.17	0.34
**cg22786465***	*LY6G5C*	downstream	0.47	0.0052
cg02733847*	*LY6G5C*	downstream	0.17	0.34
cg25769566*	*LY6G5C*	downstream	0.30	0.08
**cg24520975***	*LY6G5C*	downstream	0.38	0.03
cg11239749*	*HLA-DOB*	intronic	0.13	0.46
cg19350197	*HLA-DOB*	exonic	-0.24	0.17
cg25824217	*HLA-DPA1*	intronic	-0.62	1.02×10^{-4}
cg07306190*	*UHRF1BP1*	intronic	-0.10	0.56
cg06298701*	*NCOA4*	intronic	-0.10	0.57
cg17620335*	*NCOA4*	intronic	-0.17	0.33
cg07185131	*EHBP1*	upstream	0.06	0.72
cg01715842*	*ZDHHC7*	upstream	-0.25	0.15
cg20056908	*VAMP8*	UTR3	-0.03	0.86
**cg02652597***	*VAMP5*	upstream	-0.37	0.03
cg15059474	*BAIAP2L1*	intronic	-0.34	0.05
cg08336300*	*SESN1*	intronic	-0.04	0.81
cg17117243*	*SESN1*	intronic	-0.26	0.14
cg07128416	*CFAP44*	upstream	-0.03	0.89
cg07054641	*CFAP44*	upstream	-0.15	0.39
cg20138861	*GPR160*	intronic	0.05	0.78
**cg10165864***	*PDK1*	upstream	-0.38	0.03
cg16797009*	*PDK1*	downstream	-0.05	0.77
cg20240347	*PIK3C2B*	downstream	-0.11	0.55

cg20240347*	*MDM4*	upstream	0.10	0.59
**cg15199181***	*NUCKS1*	upstream	-0.62	1.01×10^{-4}
**cg14893161***	*PM20D1*	UTR5	-0.57	4.81×10^{-4}
**cg07167872***	*PM20D1*	upstream	-0.67	1.45×10^{-5}
**cg24503407***	*PM20D1*	upstream	-0.69	6.00×10^{-6}
**cg07157834***	*PM20D1*	upstream	-0.77	1.17×10^{-7}

* represents association with the same direction of effect compared to the association of the corresponding gene-CpG site in blood tissue; bold represents those significant association at $P<0.05$ with the same direction of effect

Supplementary Table 6. Canonical pathways, diseases, bio functions and networks associated with the genes of interest.

Top canonical pathways	Top diseases and disorders	Molecular and cellular functions	Top networks
Cell Cycle: G2/M DNA Damage Checkpoint Regulation;<br>Cancer Drug Resistance By Drug Efflux	Developmental Disorder;<br>Endocrine System Disorders;<br>Hereditary Disorder;<br>Neurological Disease;<br>Organismal Injury and Abnormalities	Cell Death and Survival;<br>Lipid Metabolism;<br>Molecular Transport;<br>Small Molecule Biochemistry;<br>Cellular Development	Cell Death and Survival, Cell Morphology, Hematological Disease;<br>Developmental Disorder, Hereditary Disorder, Neurological Disease;<br>Infectious Diseases, Post-Translational Modification, Respiratory Disease;<br>Cell Cycle, Cellular Movement, Connective Tissue Development and Function

Supplementary Table 7. Significant three-way associations with inconsistent direction of effect for methylation-gene expression-prostate cancer risk pathway

CpG site	Chr	Position	Associated gene	Classification	DNA methylation and prostate cancer risk		DNA methylation and gene expression		Gene expression and prostate cancer risk	
					OR	*P* value	Association coefficient	Association *P* value	OR	P value
cg20056908	2	85808945	*VAMP8*	UTR3	1.09	1.63×10^{-11}	0.20	3.03×10^{-14}	0.66	1.37×10^{-3}
cg13197078	6	31963919	*C4B*	intronic	0.92	1.04×10^{-7}	-0.13	3.24×10^{-6}	0.92	3.65×10^{-8}
cg15059474	7	97990184	*BAIAP2L1*	intronic	0.92	2.64×10^{-9}	0.11	9.72×10^{-5}	2.21	5.86×10^{-17}
cg06298701	10	51566673		intronic	1.72	4.08×10^{-101}	-0.08	1.90×10^{-3}		
cg17620335	10	51566824		intronic	1.22	4.44×10^{-34}	-0.08	2.51×10^{-3}	3.80	1.39×10^{-22}
cg01330312	10	51567670	*NCOA4*	intronic	1.36	2.62×10^{-90}	-0.12	1.28×10^{-5}		

Supplementary Figure 1. DNA methylation prediction model performance (R^2) in FHS and WHI datasets

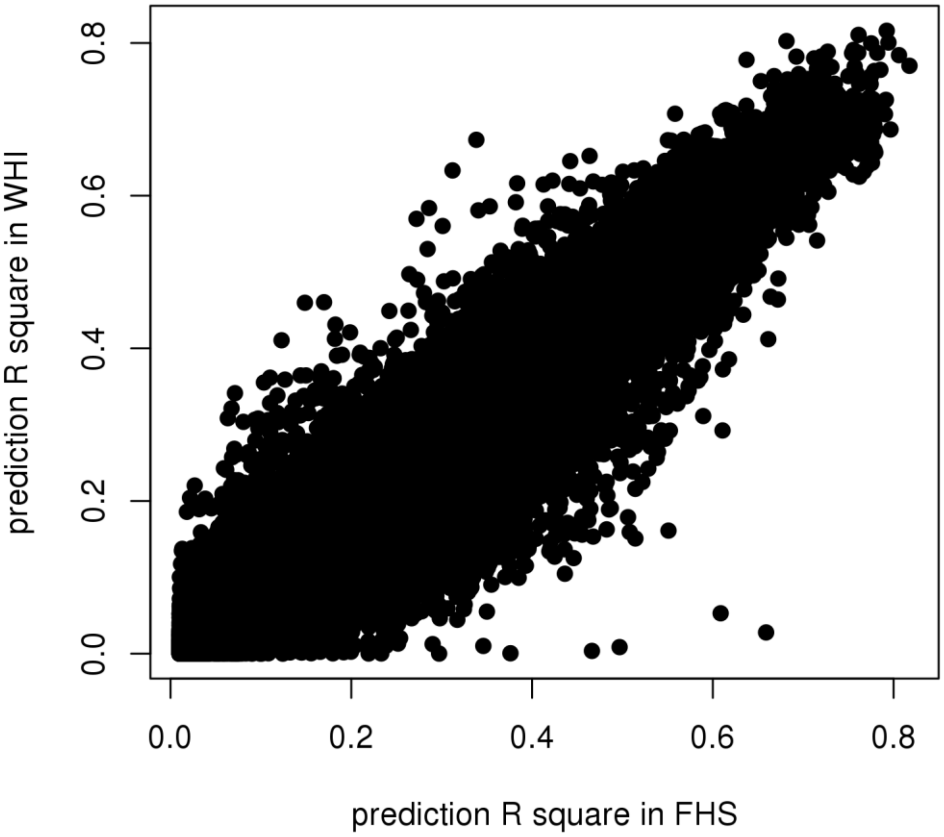

DNA methylation that could be predicted well in FHS also tended to be predicted well in WHI (a correlation coefficient of 0.96 for R^2 in two data sets

Supplementary Figure 2. Enrichment of prostate cancer associated CpG sites in regions overlapping H3K4me1 markers from the consolidated Roadmap Epigenomics data

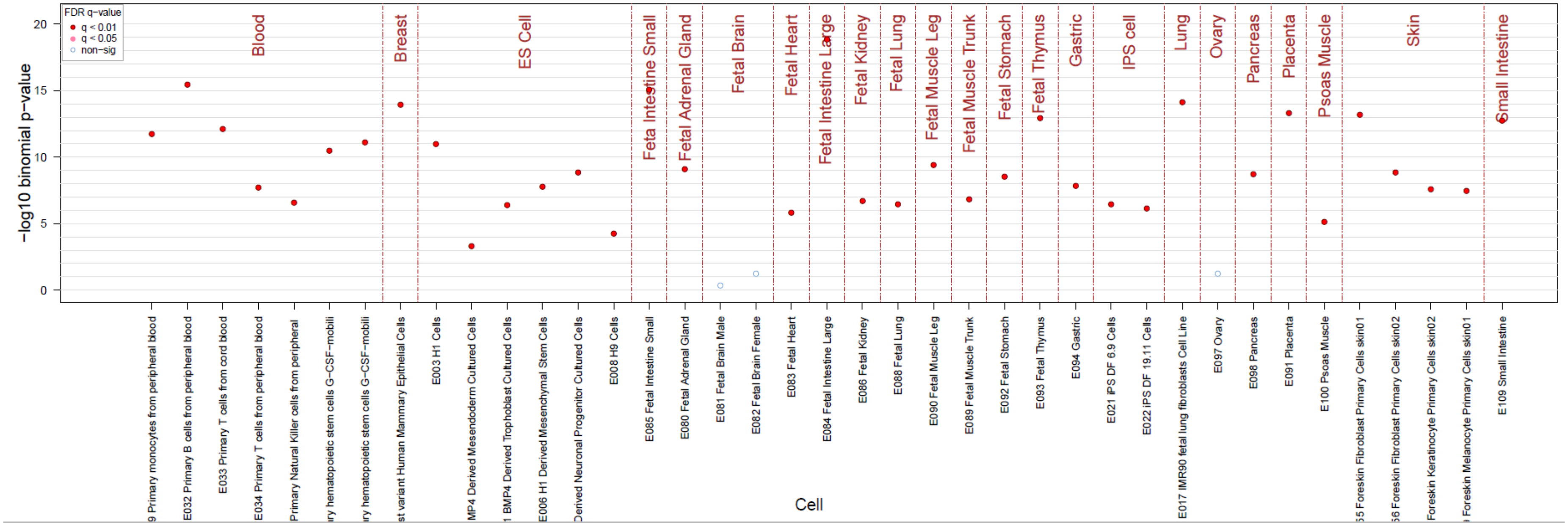

There tends to be an overlap of their positions with regions containing lysine 4 mono-methylated H3 histone (H3K4me1) markers across 38 of 39 cell types included in the consolidated Roadmap Epigenomics Project, including blood tissues

Supplement Notes:

Funding Acknowledgements

CRUK and PRACTICAL consortium

This work was supported by the Canadian Institutes of Health Research, European Commission's Seventh Framework Programme grant agreement n° 223175 (HEALTH-F2-2009-223175), Cancer Research UK Grants C5047/A7357, C1287/A10118, C1287/A16563, C5047/A3354, C5047/A10692, C16913/A6135, and The National Institute of Health (NIH) Cancer Post-Cancer GWAS initiative grant: No. 1 U19 CA 148537-01 (the GAME-ON initiative).

We would also like to thank the following for funding support: The Institute of Cancer Research and The Everyman Campaign, The Prostate Cancer Research Foundation, Prostate Research Campaign UK (now Prostate Action), The Orchid Cancer Appeal, The National Cancer Research Network UK, The National Cancer Research Institute (NCRI) UK. We are grateful for support of NIHR funding to the NIHR Biomedical Research Centre at The Institute of Cancer Research and The Royal Marsden NHS Foundation Trust. The Prostate Cancer Program of Cancer Council Victoria also acknowledge grant support from The National Health and Medical Research Council, Australia (126402, 209057, 251533, , 396414, 450104, 504700, 504702, 504715, 623204, 940394, 614296,), VicHealth, Cancer Council Victoria, The Prostate Cancer Foundation of Australia, The Whitten Foundation, PricewaterhouseCoopers, and Tattersall's. EAO, DMK, and EMK acknowledge the Intramural Program of the National Human Genome Research Institute for their support.

Genotyping of the OncoArray was funded by the US National Institutes of Health (NIH) [U19 CA 148537 for ELucidating Loci Involved in Prostate cancer SuscEptibility (ELLIPSE) project and X01HG007492 to the Center for Inherited Disease Research (CIDR) under contract number HHSN268201200008I]. Additional analytic support was provided by NIH NCI U01 CA188392 (PI: Schumacher).

Funding for the iCOGS infrastructure came from: the European Community's Seventh Framework Programme under grant agreement n° 223175 (HEALTH-F2-2009-223175) (COGS), Cancer Research UK (C1287/A10118, C1287/A 10710, C12292/A11174, C1281/A12014, C5047/A8384, C5047/A15007, C5047/A10692, C8197/A16565), the National Institutes of Health (CA128978) and Post-Cancer GWAS initiative (1U19 CA148537, 1U19 CA148065 and 1U19 CA148112 - the GAME-ON initiative), the Department of Defence (W81XWH-10-1-0341), the Canadian Institutes of Health Research (CIHR) for the CIHR Team in Familial Risks of Breast Cancer, Komen Foundation for the Cure, the Breast Cancer Research Foundation, and the Ovarian Cancer Research Fund.

BPC3

The BPC3 was supported by the U.S. National Institutes of Health, National Cancer Institute (cooperative agreements U01-CA98233 to D.J.H., U01-CA98710 to S.M.G., U01-CA98216 to E.R., and U01-CA98758 to B.E.H., and Intramural Research Program of NIH/National Cancer Institute, Division of Cancer Epidemiology and Genetics).

CAPS

CAPS GWAS study was supported by the Cancer Risk Prediction Center (CRisP; www.crispcenter.org), a Linneus Centre (Contract ID 70867902) financed by the Swedish Research Council, (grant no K2010-70X-20430-04-3), the Swedish Cancer Foundation (grant no 09-0677), the Hedlund Foundation, the Soederberg Foundation, the Enqvist Foundation, ALF funds from the Stockholm County Council. Stiftelsen Johanna Hagstrand och Sigfrid Linner's Minne, Karlsson's Fund for urological and surgical research.

PEGASUS

PEGASUS was supported by the Intramural Research Program, Division of Cancer Epidemiology and Genetics, National Cancer Institute, National Institutes of Health.

Reference

1 Yang, J. *et al.* Conditional and joint multiple-SNP analysis of GWAS summary statistics identifies additional variants influencing complex traits. *Nature genetics* **44**, 369-375, S361-363, doi:10.1038/ng.2213 (2012).
