# Supplementary material for: An integrative multi-omics analysis to identify candidate DNA methylation biomarkers related to prostate cancer risk
Source: Nat Commun. 2020 Aug 6;11:3905. doi: 10.1038/s41467-020-17673-9 (PMC7413371; doi:10.1038/s41467-020-17673-9)
Supplement: Supplementary file 2 — Description of Additional Supplementary Files [file 41467_2020_17673_MOESM2_ESM.pdf]

Description of Additional Supplementary Files

File Name: Supplementary Data 1
Description: Six hundred eighty-one methylation-prostate cancer associations potentially influenced by prostate cancer risk variantsa for CpG sites at prostate cancer risk loci

File Name: Supplementary Data 2
Description: CpG sites showing a consistent association with prostate cancer risk at $P < 0.05$ in the UK Biobank data

File Name: Supplementary Software 1
Description: These include the code used to develop genetic prediction models for DNA methylation levels, code used to determine associations between genetically predicted DNA methylation levels and prostate cancer risk, as well as a readme file.
