# Supplementary material for: An integrative multi-omics analysis to identify candidate DNA methylation biomarkers related to prostate cancer risk
Source: Nat Commun. 2020 Aug 6;11:3905. doi: 10.1038/s41467-020-17673-9 (PMC7413371; doi:10.1038/s41467-020-17673-9)
Supplement: Supplementary file 6 — Reporting Summary [file 41467_2020_17673_MOESM6_ESM.pdf]

nature research

Corresponding author(s): Jirong Long

Last updated by author(s): Jun 6, 2020

Reporting Summary

Nature Research wishes to improve the reproducibility of the work that we publish. This form provides structure for consistency and transparency in reporting. For further information on Nature Research policies, see Authors & Referees and the Editorial Policy Checklist .

Statistics

For all statistical analyses, confirm that the following items are present in the figure legend, table legend, main text, or Methods section.

n/a	Confirmed	
☐	☒	The exact sample size (*n*) for each experimental group/condition, given as a discrete number and unit of measurement
☐	☒	A statement on whether measurements were taken from distinct samples or whether the same sample was measured repeatedly
☐	☒	The statistical test(s) used AND whether they are one- or two-sided<br>*Only common tests should be described solely by name; describe more complex techniques in the Methods section.*
☐	☒	A description of all covariates tested
☐	☒	A description of any assumptions or corrections, such as tests of normality and adjustment for multiple comparisons
☐	☒	A full description of the statistical parameters including central tendency (e.g. means) or other basic estimates (e.g. regression coefficient) AND variation (e.g. standard deviation) or associated estimates of uncertainty (e.g. confidence intervals)
☐	☒	For null hypothesis testing, the test statistic (e.g. *F*, *t*, *r*) with confidence intervals, effect sizes, degrees of freedom and *P* value noted<br>*Give P values as exact values whenever suitable.*
☒	☐	For Bayesian analysis, information on the choice of priors and Markov chain Monte Carlo settings
☒	☐	For hierarchical and complex designs, identification of the appropriate level for tests and full reporting of outcomes
☐	☒	Estimates of effect sizes (e.g. Cohen's *d*, Pearson's *r*), indicating how they were calculated

Our web collection on statistics for biologists contains articles on many of the points above.

Software and code

Policy information about availability of computer code

Data collection	No software was used
Data analysis	R version 3.1.2; Plink v1.07; GCTA Version 1.26.0; MetaXcan (version 0.2.5); eFORGE v1.2; ANNOVAR; IPA software (version 51963813);<br><br>For the model building and association analyses, we used standard framework of PrediXcan and S-PrediXcan. Source codes for such analyses have been deposted at https://github.com/hakyimlab

For manuscripts utilizing custom algorithms or software that are central to the research but not yet described in published literature, software must be made available to editors/reviewers. We strongly encourage code deposition in a community repository (e.g. GitHub). See the Nature Research guidelines for submitting code & software for further information.

Data

Policy information about availability of data

All manuscripts must include a data availability statement. This statement should provide the following information, where applicable:

- Accession codes, unique identifiers, or web links for publicly available datasets
- A list of figures that have associated raw data
- A description of any restrictions on data availability

The OncoArray genotype data and relevant covariate information (i.e. ethnicity, country, principal components, etc.) for prostate cancer study are available in dbGAP (Accession #: phs001391.v1.p1). In total, 47 of the 52 OncoArray studies, encompassing nearly 90% of the individual samples, are available. The previous meta-analysis summary results and genotype data are currently available in dbGaP (Accession #: phs001081.v1.p1). The datasets of FHS Offspring Cohort and WHI are publicly available via dbGaP (www.ncbi.nlm.nih.gov/gap): dbGaP Study Accession: phs000342 and phs000724 for FHS, and phs000315, phs000675 and phs001335 for WHI. TCGA data can be accessed through the Genomic Data Commons Data Portal.

Field-specific reporting

Please select the one below that is the best fit for your research. If you are not sure, read the appropriate sections before making your selection.

- [x] Life sciences
- [ ] Behavioural & social sciences
- [ ] Ecological, evolutionary & environmental sciences

For a reference copy of the document with all sections, see nature.com/documents/nr-reporting-summary-flat.pdf

Life sciences study design

All studies must disclose on these points even when the disclosure is negative.

Sample size	79,194 cases and 61,112 controls of European ancestry included in the PRACTICAL, CRUK, CAPS, BPC3 and PEGASUS consortia; Currently, this is the largest available dataset for prostate cancer genetic study in Europeans;<br>genetic and white blood cell DNA methylation data from the FHS Offspring Cohort (N=1,595);<br>data from The Women's Health Initiative (WHI) (N=883);<br>prostate cancer patients in The Cancer Genome Atlas (TCGA; N=34);
Data exclusions	For FHS and WHI datasets, we restricted analyses to Europeans only. This is per-established considering that for FHS/WHI, a majority of subjects are Europeans;
Replication	We used data from the UK Biobank to validate the associations identified for genetically predicted DNA methylation levels.
Randomization	Not relevant to this study as this is an epidemiological study. We carefully controlled for relevant covariates in relevant analyses.
Blinding	Not relevant to this study as this is an epidemiological study.

Reporting for specific materials, systems and methods

We require information from authors about some types of materials, experimental systems and methods used in many studies. Here, indicate whether each material, system or method listed is relevant to your study. If you are not sure if a list item applies to your research, read the appropriate section before selecting a response.

Materials & experimental systems

n/a	Involved in the study	
x		Antibodies
x		Eukaryotic cell lines
x		Palaeontology
x		Animals and other organisms
	x	Human research participants
x		Clinical data

Methods

n/a	Involved in the study	
x		ChIP-seq
x		Flow cytometry
x		MRI-based neuroimaging

Human research participants

Policy information about studies involving human research participants

Population characteristics	79,194 PrCa cases and 61,112 controls of European ancestry in the PRACTICAL, CRUK, CAPS, BPC3 and PEGASUS consortia. In brief, 46,939 PrCa cases and 27,910 controls were genotyped using OncoArray, which included 570,000 SNPs (http://epi.grants.cancer.gov/oncoarray/). Also included were data from several previous PrCa GWAS of European ancestry: UK stage 1 and stage 2, CaPS 1 and CaPS 2, BPC3, NCI PEGASUS, and iCOGS.<br><br>FHS Offspring Cohort includes a sample of 5,124 men and women, consisting of the offspring of the Original FHS Cohort and their spouses. At entry, 3,483 men and 2,641 women with age less than 70 were included. In this study we used data of 1,595 Europeans.<br><br>The Women's Health Initiative originally enrolled 161,808 women aged 50-79 between 1993 and 1998. In this study we used data of 883 unrelated healthy female participants of European descent.<br><br>The UK Biobank is a prospective cohort study with deep genetic, physical and health data collected on ~500,000 individuals across the United Kingdom from 2006-2010.<br><br>The Cancer Genome Atlas is a landmark cancer genomics program, molecularly characterized over 20,000 primary cancer and matched normal samples spanning 33 cancer types. In this study, we used data of 34 PrCa patients with adjacent normal prostate tissue data available.
Recruitment	Most of the studies contributing to the OncoArray were case-control studies primarily based in either the United States or Europe. In total, 52 new studies provided core data on disease status, age at diagnosis (age at observation or questionnaire for controls), family history of PrCa, and clinical factors for cases (for example, PSA at diagnosis and Gleason score) for 48,455 PrCa cases and 28,321 disease-free controls. Previous GWAS contributed an additional 32,255 PrCa cases and 33,202 disease-free controls of European ancestry to the overall meta-analysis
Ethics oversight	All studies participating the PRACTICAL consortia were approved by the appropriate ethics committees (as described in the references for each study listed in Supplementary Table 1 of the paper PMID: 29892016), and informed consent was obtained from all participants.

Note that full information on the approval of the study protocol must also be provided in the manuscript.
